# *Theileria annae* (syn. *Babesia microti*-like) infection in dogs in NW Spain detected using direct and indirect diagnostic techniques: clinical report of 75 cases

**DOI:** 10.1186/s13071-015-0825-2

**Published:** 2015-04-10

**Authors:** Guadalupe Miró, Rocío Checa, Andrea Paparini, Nieves Ortega, José Luís González-Fraga, Alex Gofton, Adrián Bartolomé, Ana Montoya, Rosa Gálvez, Pedro Pablo Mayo, Peter Irwin

**Affiliations:** Department of Animal Health, Veterinary Faculty, Universidad Complutense de Madrid, Madrid, Spain; Vector- and Water-Borne Pathogen Research Group, School of Veterinary & Life Sciences, Murdoch University, Murdoch, WA Australia; Xarope Veterinary Centre, Laracha, Coruña Spain; Gran Vía Veterinary Centre, Carballo, Coruña Spain; Hospital Veterinario Nacho Menes, Gijón, Asturias Spain

**Keywords:** *Theileria annae*, *Babesia microti*-like, Canine piroplasmosis, Tick-borne diseases, Dog, IFAT, PCR

## Abstract

**Background:**

In north-western Spain, piroplamosis caused by *Theileria annae* is now recognized as a serious problem because veterinarians, despite being aware of the clinical signs of piroplasmosis, lack the necessary information on its epidemiology or specific diagnostic tools for its management. This, along with the fact that *T. annae* infection is also refractory to current piroplamosis treatments, prompted this study designed to assess the clinical presentation and diagnosis of this largely unknown parasitic disease in dogs.

**Methods:**

One hundred and twenty dogs in NW Spain suspected clinically of having piroplasmosis were examined and piroplasm species detected by light microscopy (LM) observation of Giemsa-stained blood smears, immunofluorescent antibody test (IFAT), and PCR plus sequencing.

**Results:**

Seventy five of the sick dogs were confirmed to be infected with *T. annae* by PCR (designated “true infection cases”). Intraerythrocytic ring-shaped bodies morphologically compatible with small piroplasms were observed by LM in 59 (57 true infections) of the 120 blood samples. Anti-*Babesia* antibodies were detected by IFAT in 59 of the 120 sera (55 of which were “true infections”). Using PCR as the reference method, moderate agreement was observed between positive LM *vs* PCR and IFAT *vs* PCR results (kappa values: 0.6680 and 0.6017, respectively). Microscopy examination and IFAT were moderately sensitive in detecting the pathogen (76% and 73.3%, respectively). In the 75 cases of “true infection”, the most common clinical signs observed were pale mucous membranes, anorexia and apathy. Blood cell counts consistently revealed severe regenerative anaemia and thrombocytopenia in dogs with piroplasmosis due to *T. annae*. Young dogs (≤3 year) (p = 0.0001) were more susceptible to the disease.

**Conclusion:**

Microscopy showed moderate diagnostic sensitivity for acute *T. annae* infection while IFAT-determined antibody titres were low (1/64 to 1/128). The infecting species should be therefore confirmed by molecular tests. Our results suggest that the disease affects dogs in regions of Spain bordering the endemic Galicia area where this piroplasm has not been previously reported (Asturias, northern Spain). Further epidemiological surveys based on serological and molecular methods are required to establish the current geographical range of *T. annae* infection.

## Background

*Theileria annae (syn. Babesia microti-*like) is a recently recognized piroplasm that causes canine piroplasmosis along with other species of the genera *Theileria* and *Babesia* [1,2].

Historically, *Babesia* infection in dogs was identified according to the morphologic appearance of the parasite in the erythrocyte. Based on relative size, these parasites are broadly divided into two groups, large and small piroplasms. Although all large forms reported to date have been ascribed to the genus *Babesia*, small *Babesia* spp. and *Theileria* spp. cannot be distinguished by microscopy and DNA-based molecular techniques are required for an accurate identification [3,4]. Indeed, it is currently unclear if the protozoan *T. annae* is a member of the genus *Theileria* or *Babesia* [2,5]. No evidence was initially presented [2] for extra-erythrocytic infecting stages or for the absence of transovarial transmission in ticks (distinguishing features of *Theileria* spp.). Morphologically, *T. annae* resembles small piroplasms such as *Babesia gibsoni* which, phylogenetically, is considered a “true babesia”. Molecularly, however, it appears to be closer to the genetically-distinct rodent piroplasm *B. microti* (*B. microti* group) and only distantly related to “true theilerias” such as *Theileria parva* [2,6,7].

At present, 12 piroplasm species have been reported in dogs worldwide but some of these have been only detected by molecular techniques [1]. Four species have been described in Europe: *Babesia canis, Babesia vogeli, B. gibsoni* and *T. annae*.

*Babesia canis* is endemic in temperate regions and is the most common species reported in Europe (northern Spain, Portugal, France, central Europe and Eastern Europe). *Babesia vogeli* and *B. gibsoni* are widely distributed across both Old and New World continents [8,9]. In Europe, *B. vogeli* has been described in the Mediterranean basin, whereas *B. gibsoni* only occasionally appears in Europe [10], mainly as the consequence of introduced infected dogs from endemic areas (Asia, United States and Australia) [8].

Studies in the United States and Australia have indicated that direct dog to dog transmission (in American Pit bull terriers and other fighting dogs) is likely and this could be the main mode of transmission outside Asia for *B. gibsoni* [11,12].

*T. annae* was first described in 2000 in a dog from Germany that had travelled to the Pyrenees [2]. Today, the infection is considered endemic in NW Spain (Galicia) [5,13]. Using molecular techniques *T. annae* has been also detected in Spanish regions outside Galicia, such as Barcelona [14], and in other countries including NW Portugal [15], Croatia [16], US [12] and Sweden [17]. However, in most cases the travel history of the dogs was unknown. Other authors have reported cases of *T. annae* infecting foxes in Spain [18], Portugal [19], Italy [20], Croatia [21], Canada [22] and the US [23]. Among all piroplasm species reported in Europe, *T. annae* seems to show the greatest preference for foxes. Thus, *T. annae* has been detected in red foxes in Spain and Portugal at prevalences from 14% to 69.2%, respectively; while *B. canis* has been only occasionally identified in these animals [19]. To date, there are no available data regarding clinical impacts on foxes.

The transmission vector of *T. annae* is presently unknown. *Ixodes hexagonus* has been proposed as a likely candidate because endemic areas of *T. annae* infection closely match its distribution range [18,24]. However, *T. annae* DNA has been observed in both *I. hexagonus* and *I. ricinus,* though no data exist to substantiate their competence as vectors for *T. annae*.

*T. annae*-infected dogs show severe clinical signs and clinicopathological abnormalities resembling those of other piroplasm infections such as fever, pale mucous membranes or haemoglobinuria [25]. Despite morphologic differences, *T. annae* is often ascribed by veterinarians to other *Babesia* spp. mainly *B. canis*. Currently, there are no diagnostic tools to distinguish between the different piroplasms in routine veterinary practice and their detection by microscopy in red blood cells is still the only method available to practitioners. In laboratory settings, the immunofluorescent antibody test (IFAT) is the most widely used test for a serology diagnosis and is considered highly sensitive and moderately specific to detect chronic infection and subclinical infection in carriers [4,26,27]. The polymerase chain reaction (PCR) is a sensitive and specific diagnostic test widely employed to diagnose canine babesiosis. When PCR is combined with sequencing, species-specific primers/probes, or restriction fragment length polymorphism (RFLP) analysis it can be used to detect infected dogs with low parasitaemia levels and to identify parasites [12,28,29]. However, the literature lacks widespread serological and/or molecular surveys of *T. annae* infection. Similarly, comparative methodological studies on the available diagnostic procedures are limited.

The present study was designed to examine the clinical picture of *T. annae* infection in dogs in NW Spain and assess how best to diagnose this largely unknown disease.

## Methods

### Sample and data collection

Over the period June 2012 to January 2014, dogs from several Veterinary Clinics in NW Spain were tested for *Theileria annae.* Inclusion criteria for the enrolment of dogs were clinical signs suggestive of piroplasmosis such as: pale mucous membranes, apathy, anorexia, orangey faeces, fever, weight loss, or haematuria. In all dogs the presence of large piroplasms (*B. canis* or *B. vogeli*) was first ruled out via microscopy by collaborating practitioners.

All participating dogs were subjected to a clinical examination and blood collection. From each dog, a 4.5 ml blood sample was obtained by cephalic venipuncture and 1.5 ml of the collected blood placed in two EDTA tubes: a 1 ml tube used for full blood counts and blood smears; and a 0.5 ml tube used to detect *T. annae* by genomic DNA isolation, PCR and sequencing. Also, 3 ml of the collected blood samples were placed in tubes without anticoagulant for biochemical profiles and antibody testing. All blood samples were kept at 4°C until processing.

In the clinical file, we recorded the: identification number, age, breed, sex, weight, rural or urban living environment and travel history. Also considered were the clinical history and the specific clinical signs at the time of sampling such as changes in the colour of mucous membranes, anorexia, haematuria, fever, weight loss, splenomegaly, hepatomegaly, or lymphadenomegaly.

### Haematology and biochemistry

The following variables were determined using an automated blood analyser (Sysmex XT-2000i, Roche Diagnostics, Spain): red-blood-cell count (RBCC), reticulocyte count, haemoglobin concentration, haematocrit, red cell distribution width (RDW), mean corpuscular volume (MCV), mean corpuscular haemoglobin (MCH), mean corpuscular haemoglobin concentration (MCHC), leukocyte and platelet count. Differential white blood cell counts were conducted by conventional microscopy procedures. A clinical biochemical analyser (Cobas integra® 400 plus, Roche Diagnostics, Spain) was used for serum concentrations of glucose, total protein, albumin, globulin, urea and creatinine; aspartate aminotransferase (AST) activity, alanine aminotransferase (ALT) activity, glutamyltransferase (GGT) activity, creatinine kinase (CK), alkaline phosphatase (ALP) activity and total, direct and indirect bilirubin concentrations. All tests were performed using standard techniques. Platelet numbers and hepatic enzyme activities could not always be determined due to platelet aggregation or haemolysis.

### Microscopic detection of the parasite

Giemsa-stained thin blood smears were examined by light microscopy (LM) to detect small intraerythrocyte ring-shaped bodies compatible with *T. annae*. The smears were air dried, fixed in absolute methanol for 5 min, stained using 20% Giemsa and then observed using a 1000× magnification objective under immersion oil. All samples were examined by the same technician.

### Serum antibodies

Anti-*Babesia* antibodies were detected by the immunofluorescent antibody test (IFAT) using a commercially available antigen kit (MegaScreen® FLUO BABESIA microti, Austria). Fixed erythrocytes infected with *Babesia microti* were used as antigen. The IFAT was performed according to the manufacturer’s instructions using a cut-off = 1:64 to denote seropositivity. Positive sera were further tested in a serial dilution series (from1:32). Slides were examined by the same reader under a fluorescence microscope.

Serological testing for the most prevalent CVBD present in Spain [30], *Leishmania infantum* and *Ehrlichia canis,* was also performed by IFAT.

In the *L. infantum* test, specific antibodies were detected against in-house cultured promastigotes and anti-*Leishmania*-specific immunoglobulin G (IgG) antibodies were detected as described previously [31] using a cut-off =1:100 to denote seropositivity. The serial dilutions prepared were 1/25, 1/50, 1/100, 1/200, 1/400, 1/800 and 1/1600.

IFAT for anti-*E. canis* antibodies was only performed in 75 dogs in which *T. annae* was PCR confirmed. For this test, a local strain was used as antigen and a cut-off =1:80 was taken to denote seropositivity.

### DNA isolation and PCR-RFLP

Genomic DNA was isolated from peripheral whole blood (100 μl) using the QIAamp® DNA blood micro kit (QIAGEN®, USA) as described by the manufacturer. The extracted DNA was eluted in molecular-grade water (70 μl) and stored at −20°C until further use. DNA quality was quantified fluorometrically using the Qubit® system (Life Technologies, USA). Blood-DNA was screened for piroplasms using PCR-based assays targeting the small subunit ribosomal RNA gene (18S rDNA). The implemented assays included a shorter nested PCR (850 bp; primers BT F1/R1 followed by BT F2/R2) [28].

PCR products were run on a 1% agarose gel containing SYBR Safe Gel Stain (Invitrogen, USA), and visualized with a dark reader trans-illuminator (Clare Chemical, USA). PCR products corresponding to the expected length were excised, and sequenced using an ABI Prism Terminator Cycle Sequencing kit (Applied Biosystems, USA) in an Applied Biosystem 3730 DNA Analyzer.

### Phylogenetic analysis

Phylogenetic analysis was conducted on the sequences obtained during the present study and additional piroplasms sequences available in GenBank.

Sequence chromatogram files were analyzed by FinchTV 1.4 (http://www.geospiza.com), and imported into Geneious Pro V. 7.1.5 (Biomatters, Auckland, NZ), for editing, assembly and alignments. Alignments obtained by MAFFT v7.017 [32] and MUSCLE [33] were trimmed manually and trees were reconstructed using the Geneious FastTree plugin [34]. When applicable, alignments were curated by Gblocks [35], remotely [36] with the low-stringency set of options selected. *Cardiosporidium cionae* was used as an outgroup based on previous recommendations [37].

### Tick collection and identification

Ticks were obtained from dogs observed to have ticks in the clinical examination and stored in 70% ethanol for identification to species level, sexing and staging using morphological keys [38].

### Statistical analysis

Results were analysed using the statistics package SAS version 9.4. IFAT and microscopy results were compared with molecular results using McNemar’s test, simple Kappa coefficient and Wilcoxon scores (Rank Sums). PCR and sequencing was used as the gold standard reference method to identify small piroplasms [29,39]. Sensitivity and specificity are provided for each of the techniques used. Sensitivity was calculated as the number of LM or IFAT positive results divided by the number of PCR positive results, and specificity as the number of LM or IFAT negative results divided by the total number of PCR negative results. According to the central limit theorem, our sample was sufficiently large (N > 30) for the use of parametric tests. Relationships between *T. annae* infection and the remaining categorical variables were assessed using the Chi-squared test and between *T. annae* infection and continuous variables by the Student t-test. Significance was set at *p ≤ 0.05*.

## Results

### Molecular diagnosis

Inclusion criteria for this study were met by 120 dogs. In 75 of these dogs (62.5%), *T. annae* infection was confirmed by PCR and sequencing, these animals are hereafter referred to as “true cases of *T. annae* infection”. In 15 of the dogs enrolled, *Babesia gibsoni* was PCR-detected in 3 and *B. canis* in 12*.*

When we compared our 75 sequencing results with existing GenBank entries, the sequences obtained were identified as *T. annae* in BLAST searches. All the sequences obtained were above 98% identical to *T. annae*. Several iterative bioinformatics steps were implemented for careful validation of the input data. Inclusion in the final subset used for the phylogenetic reconstruction was based on chromatogram quality, length, specificity and position of the sequence within the alignment. After a preliminary selection, 43 sequences with lengths ranging from 231 to 770 bp (Mean: 617.7; Std Dev: 125.4) were processed further. BLAST-searches returned hits for canine piroplasms and members of the *B. microti* group. After trimming, two un-curated alignments were obtained and used for the phylogenetic reconstructions (575 bp, 93 sequences, 83.9% pairwise identity and 223 bp, 104 sequences, 91.8% pairwise identity). In particular, while the shorter alignment included all the sequences from the present study, in the longer only the longest sequences were retained. Regardless of the alignment length and implementation of the optional curation step (by Gblocks), all sequences obtained during the present study clearly grouped with either canine babesias (e.g., *B. canis*, *B. gibsoni*) or within the *B. microti* group (e.g., *T. annae* isolate Dog#8, Acc. No. JX454779).

### Microscopy and serology results related to PCR results

**Figure 1:**
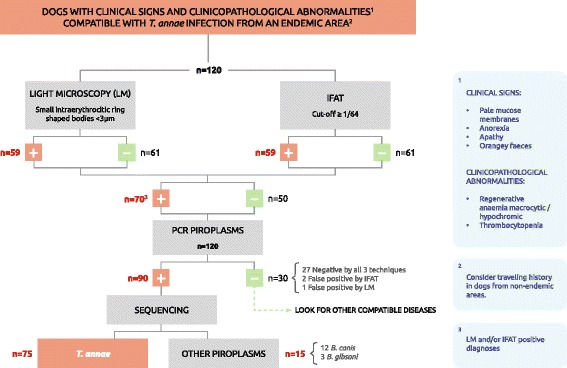
**Decision tree for the diagnostic approach to dogs with suspected clinical signs and/or clinicopathological abnormalities consistent with** ***T. annae*** **infection.** Abbreviations: IFAT = immunofluorescence antibody test, LM = light microscopy, PCR = polymerase chain reaction.

**Figure 2:**
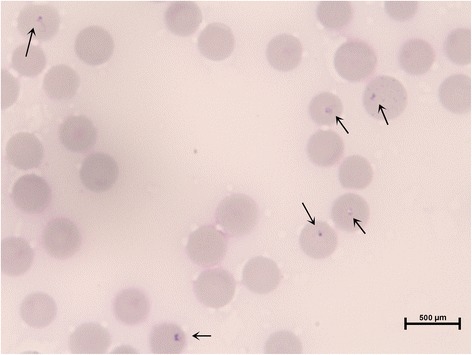
**Intraerythrocytic ring-shaped bodies, morphologically compatible with** ***T. annae*** **.** Giemsa stained blood smear (x1000).

The results obtained for the three diagnostic techniques employed are provided in Figure 1. Intraerythrocytic ring-shaped bodies, morphologically compatible with small piroplasms (Figure 2) were detected by LM in 59 of the 120 blood samples (49.2%), two of which were confirmed as false positives. In one of these false positives, no piroplasm DNA was detected by PCR while the other case was sequenced as *B. canis*.

Anti-*Babesia* antibodies were detected by IFAT in 59 of the 120 dogs (49.2%), four of which were confirmed as false positives. In two of these, no piroplasm DNA was detected and in the remaining two, the presence of *B. canis* was observed. Antibody titres were 1/64 to 1/1024 and distributed as follows: 1/64 (n = 27), 1/128 (n = 17), 1/256 (n = 4), 1/512 (n = 3), 1/1024 (n = 5), and 1/2048 (n = 3).

We observed good agreement between a positive LM and PCR result, and between a positive IFAT and PCR result (Tables 1 and 2). Kappa values indicated moderate agreement in both cases (0.6680 and 0.6017, respectively) though better agreement was observed between LM and PCR than between IFAT and PCR. Sensitivities and specificities were 76% and 95.6% for LM and 77.3% and 91.1% for IFAT, respectively

**Table 1:** **Correlation between LM and PCR used to detect** ***T. annae*** **infection**

		**PCR**		
		**Negative**	**Positive**	**Total**
LM	NEGATIVE	43	18	61
	POSITIVE	2	57	59
	TOTAL	45	75	120

Kappa 0.6680, specificity 95.56%, sensitivity 76%, positive predictive values (PPV) 96.61%, negative predictive values (NPV) 70.49%.

**Table 2:** **Correlation between IFAT and PCR used to detect** ***T. annae*** **infection**

		**PCR**		
		**Negative**	**Positive**	**Total**
IFAT	NEGATIVE	41	20	61
	POSITIVE	4	55	59
	TOTAL	45	75	120

Kappa 0.6017, specificity 91.11%, sensitivity 73.33%, positive predictive values (PPV) 93.22%, negative predictive values (NPV) 67.21%.

When comparing the use of LM plus IFAT versus PCR, a greater sensitivity was observed (85.33%) than if we used either technique on its own. However, specificity was reduced (86.66) (Table 3).

**Table 3:** **Correlation between IFAT-LM and PCR used to detect** ***T. annae*** **infection**

		**PCR**		
		**Negative**	**Positive**	**Total**
LM & IFAT	NEGATIVE	39	6	50
	POSITIVE	11	64	70
	TOTAL	45	75	120

Kappa 0.7043, specificity 86.66%, sensitivity 85.33%, positive predictive values (PPV) 91.42%, negative predictive values (NPV) 78%.

A greater number of positive PCR results was detected in samples testing LM- or IFAT negative, than negative PCR results in samples testing LM- or IFAT positive. The McNemar test indicated these were not chance discrepancies meaning that false negatives were more likely than false positives for LM (p = 0.0003) and IFAT (p = 0.0011) compared to PCR for a diagnosis of *T. annae*.

According to the Wilcoxon rank sum test, antibody titres and PCR results were positively correlated (p = 0.03) such that high antibody titres were associated with the presence of parasite DNA in the blood.

### Vector-borne diseases serology testing

Anti-*L. infantum* antibodies were detected by IFAT in 13 of the 120 dogs (10.8%). Nine of these animals (9/13) were scored positive for *T. annae* by PCR, yet antibody titres were low (1/100 in 5 dogs and 1/200 in 4 dogs). A further three of these dogs (3/13) tested PCR positive for other piroplasm species and antibody titres were also low (1/200 in 2 dogs with *B. canis* and 1/100 in 1 dog with *B. gibsoni*). These animals showed no clinical or other signs of leishmaniosis (e.g., lymphadenomegaly, cutaneous lesions, hypergammaglobulinaemia, hypoalbuminaemia). In the remaining dog showing anti-*L. infantum* antibodies (1/13), *T. annae* was not detected and clinical signs were compatible with canine leishmaniosis. In this animal the antibody titre was 1/400.

Anti-*E. canis* antibodies were not detected by IFAT in any of the 75 true infection cases.

### Clinical picture

In this section, we describe the clinical picture observed in the 75 dogs confirmed by PCR to be infected by *T. annae*. The main reasons for a visit to the veterinarian were apathy (51.4%), loss of appetite (41.6%) and weakness (19.4%). The most prevalent clinical signs observed in the physical examination were pale mucous membranes (69.9%), anorexia (73.9%) and apathy (66.6%). Other reported clinical signs were orangey faeces (12.5%), vomiting (5.5%), tachycardia (13.8%), fever (29.6%), weight loss (11.7%), haematuria (18.5%) and splenomegaly (25%). Significant differences between *T.annae*-infected and non-infected dogs were detected in the clinical signs pale mucous membranes (p = 0.0147), anorexia (p = 0.0374) and orangey faeces (p= 0.02).

The main haematological finding was regenerative anaemia in 79.6% (57/72) and non-regenerative anaemia in 6.9%. Most sick dogs had mild to severe anaemia, 80% showing less than 4.2 x 10^6^ erythrocytes/ml, 12.2 g haemoglobin/dl and 33% haematocrit. Anaemia was most often hypochromic and macrocytic. Median red blood cell counts, haemoglobin concentrations and haematocrits in infected dogs were clearly lower compared to reference values or corresponding values for the group of non-infected dogs. In addition, MCV values were significantly higher and MCHC values were significantly lower in infected compared to non-infected dogs (Table 4).

**Table 4:** **Descriptive statistics and comparative Student t-test for haematological variables recorded in** ***T. annae*** **infected (n = 75) and non-infected dogs (n =45)**

**Blood variable (normality reference range)**	**Group**	**N**	**Mean**	**SD**	**Percentiles**			**P value**
					**25** ^**th**^	**50** ^**th**^	**75** ^**th**^	
**Erythrocytes (5.50-8.50)x10** ^**6**^ **/μl**	I	72*	3.28	1.63	2.16	2.77	4.32	<0.0001
	NI	38*	4.9	2.04	3.57	5.3	6.06	
**Haematocrit (37.00-55.0)%**	I	72	27.12	11.9	18.6	23.45	36.2	0.0019
	NI	38	35.16	13.86	23.50	36.75	44.9	
**Haemoglobin (12.00-18.00)g/dl**	I	72	7.75	3.75	5	6.5	10.2	<0.0001
	NI	38	11.04	4.57	6.5	12	14.1	
**MCV (60.00-76.00)fl**	I	72	82.84	8.25	76	81.5	83.3	0.0088
	NI	38	76.02	12.99	70.1	73	75.6	
**MCHC (32.00-36.00)g/dl**	I	72	28.25	2.61	26.7	28.2	29.7	<0.0001
	NI	38	31.2	3.12	29.5	31.2	32.7	
**MCH (19.5-24.5)pg**	I	72	23.18	1.73	22.1	23.1	23.8	0.5552
	NI	38	23.59	4.08	22.1	23	23.8	
**RDW (14.00-20.25)%**	I	72	17.44	3.26	15.1	17.45	19.2	0.1385
	NI	38	16.47	3.06	14.5	14.9	18.5	
**Leukocytes (6.00-17.00)x10** ^**3**^ **/μ**	I	72	14.42	6.25	10.5	13.32	17.1	0.3649
	NI	38	12.64	11.3	7.89	10.84	14.54	
**Platelets (200–500) x10** ^**3**^ **/μl**	I	59	158.5	90.35	90	165	218	0.019
	NI	33	211.2	121.25	150	212	272	

*no data available for 3 infected and 7 non-infected dogs.

SD: standard deviation.

I = *T. annae* infected dog; NI = *T. annae* non-infected dogs.

The second most frequent haematological abnormality was thrombocytopenia (58.3%). Leukocyte counts were elevated in 18 out of 72 infected dogs (mostly with neutrophilia) and diminished in 5. Eosinopenia was observed in 23 out of 68 infected dogs. The main biochemical abnormalities detected were hyperglobulinaemia (33/65) and elevated hepatic enzyme activities (25/57). Azotaemia was observed in a few cases (7/71) yet differences were non-significant with respect to non-infected dogs.

### Epidemiological data

**Figure 3:**
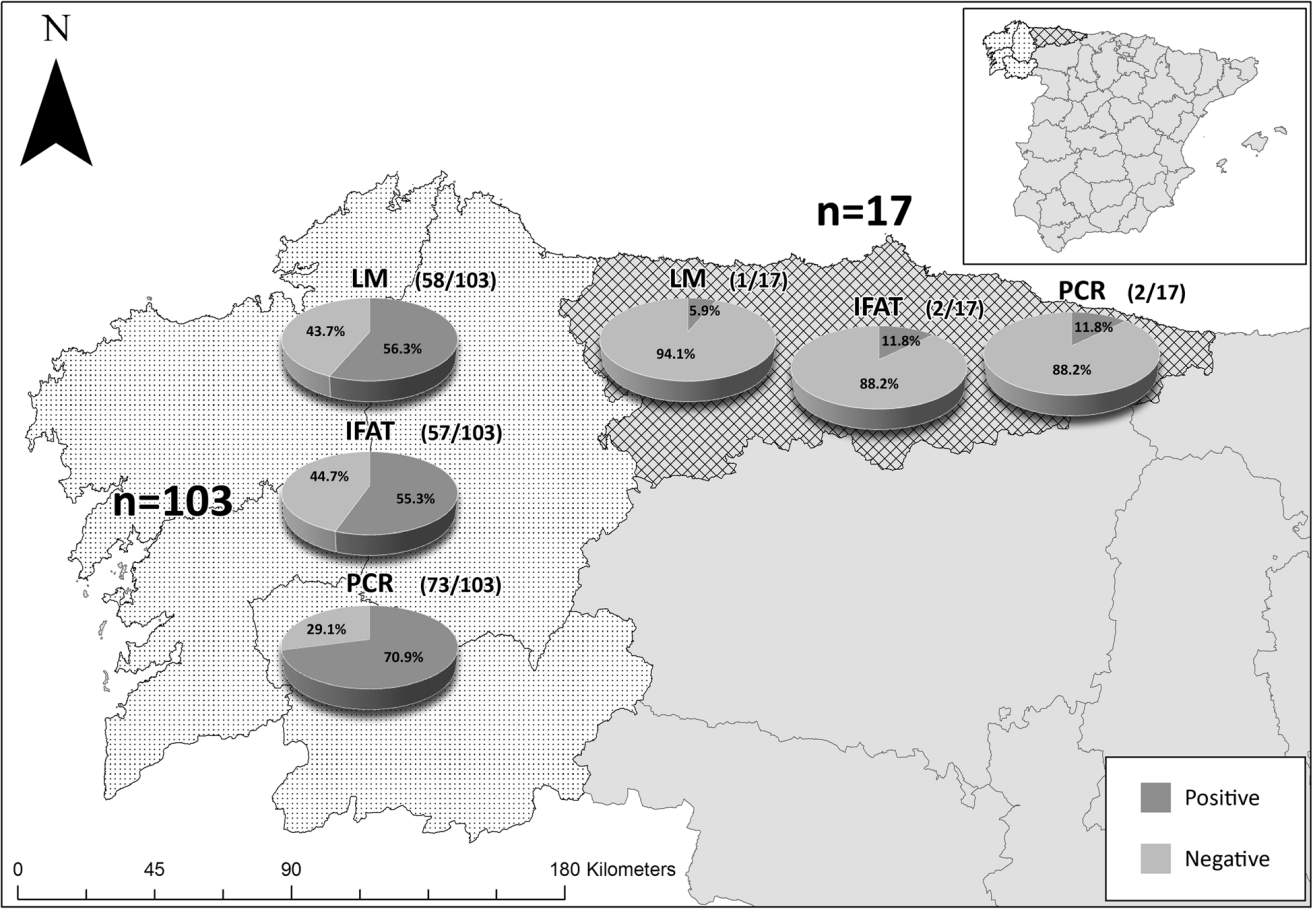
**Results obtained using each diagnostic method by study area.**

Of the 120 dogs included in this study, 103 dogs were from Galicia, 73 of which tested positive for *T. annae* infection, and 17 dogs were from a neighbouring area (Asturias), two of which tested positive for the parasite (Figure 3). One of the two PCR-confirmed cases in Asturias had never left that area.

Epidemiological data compiled for the 75 confirmed *T. annae* cases are provided in Table 5. No differences emerged according to sex or breed. However, a greater number of positive cases (81.54%) were recorded in dogs ≤ 3 years (p = 0.0001) compared to older dogs (46.8%). Significant correlations were also noted between *T. annae* infection and a small or medium dog size (≤22 Kg) (p = 0.0012) or being a hunting dog (p = 0.014).

**Table 5:** **Epidemiological data recorded in 75 dogs infected with** ***T. annae*** **confirmed by PCR and sequencing**

**Variable**		**N° total dogs**	**N° positive** ***T. annae*** **dog (%)**
Age (years)	≤3	65	53 (81.54)**
	>3	47	22 (46.81)
	unknown	8	0
Sex	Male	58	38 (65.5)
	Female	55	37 (67.2)
	unknown	7	0
Size (kg)	≤22	77	59 (76.6)*
	>22	35	16 (45.71)
	unknown	8	0
Breed	Pure breed	86	53 (61.6)
	Crossbreed	33	22 (66.6)
	unknown	1	0
Lifestyle	Hunting	75	56 (74.6)*
	Companion	28	13 (46.4)
	Guard	12	6 (50)
	unknown	5	0
Habitat	Rural	96	64 (66.6)
	Urban	24	11 (45.8)
Tick infestation	Yes	50	35 (70)
	No	49	27 (55.1)
	unknown	21	13 (10.8)
Seasonality	spring	20	14 (70)
	summer	20	10 (50)
	autumn	60	38 (63.3)
	winter	20	13 (65)

**p ≤ 0.0001; *p ≤ 0.02.

In addition, higher percentages of *T. annae* positive dogs were recorded in dogs living in rural (66.6%) than urban areas (45.8%). There was no significant correlation between seasonality and *T. annae* infection. Further, 46.6% (35/75) of *T. annae* infected dogs were found to have ticks yet no significance was detected for this risk factor (data recorded during signalment). We collected 42 ticks from 22 dogs, 15 of which were true infection cases. These ticks were identified as *Ixodes hexagonus* (50%), *Ixodes ricinus* (19%), *Dermacentor reticulatus* (16%), and *Dermacentor marginatus* (5%). Only, four nymphs could be classified to the genus level as *Ixodes spp.. Ixodes hexagonus* was identified in 10 of the 15 *T. annae* infected dogs.

## Discussion

In this study, *T. annae* infection was assessed by PCR, LM and IFAT on blood and serum samples obtained from dogs in NW Spain suspected of having piroplasmosis.

Microscopy examination is the easiest and most accessible diagnostic test, requiring a well prepared and suitably stained blood smear together with a trained observer. Our results indicate the good specificity (95.56%) and moderate sensitivity (76%) of this procedure as well as its moderate agreement with PCR. However, it should be noted that we assume that most of the dogs examined here were in the clinical phase of *T. annae* infection when the visual detection of piroplasms is easier than in animals with low parasitaemia levels due to chronic disease [1]. In effect, LM has been described as less sensitive to detect chronic and sub-clinical piroplasmosis in carrier dogs [40].

We considered the molecular approach as the gold standard method for the diagnosis of small piroplasm infections. Accordingly, using the molecular diagnostic test, a larger number of positive dogs for *T. annae* infection were detected (62.5%). Other PCR assays used to diagnose *B. gibsoni* infections have shown a high specificity and sensitivity [29,41]. In the latter studies, PCR was able to detect the parasite both at an earlier stage of infection than IFAT or LM and in the late stages of infection, when parasitaemia levels are low and Giemsa-stained thin blood smears return negative results [41]. Nevertheless, false negative PCR results have been reported in chronic babesiosis, attributed to parasite elimination from the circulating blood by the host [40]. This could determine that in the long term (up until 420 days post-infection) an infection might only be revealed (retrospectively) by serology [42].

In agreement with a previous report [43], we observed moderate agreement between our IFAT and PCR results. In contrast, discrepancies were reported by Kubelová et al. [44] for these techniques in endemic areas of canine piroplasmosis.

Serological cross-reactions between *T. annae* and *B. canis* were produced in two dogs. Cross reactions have been also reported between *B. gibsoni* and *B. canis* by other authors [45]. Most of the present dogs testing seropositive for *T. annae* (n = 59) showed low antibody titres (1/64 or 1/128). Such titres reflect an early stage of infection. For *B. canis,* it has been described that the first detectable IgG antibodies usually appear 2–3 weeks after infection [46,47]. In addition, low antibody titres could be indicative of past infection or exposure and not necessarily of present infection. A serological diagnosis is therefore a more reliable method for the detection of hidden or past infections (i.e. chronically infected carrier dogs) though acute infections may not be accurately diagnosed if this technique is performed alone. This issue, however, needs confirmation owing to a lack of serological data. It has recently been argued that the diagnosis of infection by a vector-borne pathogen in dogs can be improved by running serological and PCR based tests in parallel [48]. However, we observed no benefits of the use of both techniques over that of PCR alone. Serology is unable to distinguish between *B. canis*, *T. annae* and *B. gibsoni* infection, and blood smears cannot distinguish between *T. annae* and *B. gibsoni*. Indeed, the latter technique is also unable to discriminate *B. canis* which, though considered a large *Babesia*, can appear as having pleomorphic intermediate-sized intraerythrocyte stages.

In the current study, PCR and sequencing using universal primer sets specific for piroplasmida enabled the detection of a larger number of animals harbouring *T. annae*, compared to the other techniques (Figure 1). However, our study failed to clarify the taxonomy of *T. annae* such that more work is needed to resolve this well recognized systematics conundrum [7].

The possibility of co-infections should also be considered. In the north of Spain, summers are warm, winters cool and rainfall is evenly distributed all year round. Accordingly to that, *Rhipicephalus sanguineus* and *Phlebotomus perniciosus*, vectors frequently reported in Spain, are uncommon in this region and the prevalences of *L. infantum* and *E. canis* are lower than in the rest of Spain. The seroprevalence of *L. infantum* recorded in our study (10.8%) was higher than previously reported for NW Spain (3.7% and 4.1%) [49,50]. This discrepancy could be attributed to the sick population selected for our study rather than a cross-sectional population. An exemption might be the Orense province in NW Spain, where a prevalence of 35.6% has been observed, similar to that reported in endemic areas of Spain [50]. This prevalence was attributed by the authors to the bioclimatic characteristics of this geographical area. Moreover, in this latter region, the presence of the sandfly, *P. perniciosus* was also detected. Only one dog from Ourense, which tested negative for *L. infantum,* was enrolled in this study. Twelve of the dogs examined here showed coinfection with piroplasms (*T. annae, B. canis* or *B. gibsoni*) and *L. infantum*. The findings are similar to those in a previous study that showed high co-infection rate of *L. infantum* in the *Babesia* positive dogs from Portugal [9]. No *E. canis* antibodies were detected in our study, coinciding with the low seroprevalence (1.4%) reported for this infection in NW Spain [30].

With respect to the clinical picture, the most common clinical signs observed in the dogs included in our study were weakness, pale mucous membranes, haemoglobinuria, tachycardia, hyperthermia, tachypnea, and hepatosplenomegaly. These signs consistent with those reported previously for *T. annae* [25] and *Babesia* spp. infection [51] were to be expected because many were responsible for a clinical suspicion of piroplasmosis and were thus criteria for inclusion in our study. We observed orangey faeces in 12.5% of the infected dogs, probably due to high levels of excreted bilirubin.

Severe regenerative anaemia and thrombocytopenia were the main haematological findings observed, consistent with prior reports [13]. Regenerative anaemia was typically macrocytic/hypochromic with increased numbers of reticulocytes observed that were relatively larger than mature red blood cells. Reticulocytes are hypochromic because they have not completed haemoglobin synthesis. These haematological abnormalities have been also described by others in *T. annae* infected dogs in NW Spain [25].

The red blood cell counts, haematocrits and haemoglobin levels recorded in the present animals with suspected piroplasmosis are in agreement with those reported for 62 *T. annae* -infected dogs examined in 2003, 90% of which showed values lower than 4.46 x 10^6^ erythrocytes/μL, 10.52 g haemoglobin/dL and 31.04% haematocrit [13]. Leukocyte abnormalities have been inconsistently observed in dogs with piroplasmosis [25]. Total leukocyte counts were greater than 17 x10^3^ cells/μL in 25% of the animals examined here, and there was a trend towards neutrophilia and eosinopenia. This could be a consequence of the severe stress associated with this illness, in line with observations by other authors [13].

We only detected a few cases of azotaemia (9.8%) despite others observing its high prevalence in this disease (36%) [13,52] and suggesting its strong correlation with the likelihood of death within the first week of diagnosis [52]. High ALP activity was observed in 43.8% of the infected dogs despite reports of liver disease only in other types of piroplasmosis [53].

Our epidemiological results are in agreement with observations by García et al. [13], who mentioned that the age distribution of their study population reflected a greater risk of infection in younger animals [13]. Frequencies of infection by *Babesia* species in endemic areas have been described as inversely proportional to animal age. The correlation observed here, between an animal weight under 22 kg or a hunting type dog and a greater likelihood of *T. annae* infection, could reflect the fact that hunting dogs are usually fairly light. A large number of the *T. annae* infected dogs included in our study lived in rural areas and were infested by ticks. Other authors have reported a greater risk of *T. annae* infection in hunting dogs or dogs infested by ticks [25,54]. As also noted for other *Babesia* species [54,55], we observed no correlation between *T. annae* infection and sex or breed. García et al. [13] reported that autumn and winter were the periods when most cases of *T. annae* were observed. However, we observed no significant correlation between season and *T. annae* infection.

By PCR and sequencing, we detected three positive cases of *B. gibsoni* infection among the 120 dogs with suspected piroplasmosis. However, we have insufficient data to confirm the autochthonous nature of these cases. *B. gibsoni* has been sporadically reported in dogs travelling to endemic areas [10,14] and more epidemiological studies are needed to evaluate its presence in Spain. In contrast, *B. canis* has been often identified in dogs in northern Spain and Portugal [56] and both its diagnosis and clinical management by veterinarians in these regions are effective [8]. Despite not being included in our study, among dogs diagnosed in the participating clinics as having piroplasmosis caused by *B. canis*, 12 cases proved PCR positive. This could reflect the high prevalence of *B. canis* in NW Spain and its similar clinical signs to *T. annae* infection. Moreover, the pleomorphic nature of *B. canis* hinders its microscopy identification and a molecular confirmation is often required on which to base selection of the best treatment option. One of the two PCR-confirmed cases in Asturias (outside Galicia) had never left that area, suggesting that *T. annae* infection could be spreading to neighbouring regions. This idea requires confirmation in future studies.

## Conclusions

Currently, microscopy detection of the parasite is the most simple and rapid diagnostic method, provided it is performed by a specialized technician. This procedure shows a moderate sensitivity for the pathogen in the acute infection stage when IFAT-determined antibody titres are low. However, sensitivity increases when IFAT and LM are used together. PCR is able to detect a larger number of positive cases and confirm the species involved. For an accurate diagnosis, we would recommend an integrative approach based on epidemiological evidence, the clinical picture, LM and/or IFAT, and confirmation of the infecting species by a molecular method. We propose that the decision tree in Figure 1 may be useful for clinically managing *T. annae* infection in endemic regions or in dogs travelling to an endemic area. With regards to the possibility that this small piroplasm could spread across northern Spain, we fear that bordering areas of Galicia with similar climate conditions could be already affected. This concern determines a need for larger epidemiological surveys in which molecular and serological methods are used to detect dogs with chronic or subclinical infection. The data emerging from such studies will serve to more reliably establish the current prevalence of *T. annae* infection in northern mainland Spain. Finally, our study does not clarify the systematics of *T. annae* such that we recommend more work in this area.
